# Carbapenem-Resistant Enterobacteriaceae Bloodstream Infection Treated Successfully With High-Dose Meropenem in a Preterm Neonate

**DOI:** 10.3389/fphar.2020.566060

**Published:** 2020-09-11

**Authors:** Yue-E Wu, Hai-Yan Xu, Hai-Yan Shi, John van den Anker, Xiao-Yu Chen, Wei Zhao

**Affiliations:** ^1^ Department of Clinical Pharmacy, Key Laboratory of Chemical Biology (Ministry of Education), School of Pharmaceutical Sciences, Cheeloo College of Medicine, Shandong University, Jinan, China; ^2^ Department of Neonatology, The First Affiliated Hospital of Shandong First Medical University & Shandong Provincial Qianfoshan Hospital, Jinan, China; ^3^ Department of Clinical Pharmacy, Clinical Trial Center, The First Affiliated Hospital of Shandong First Medical University & Shandong Provincial Qianfoshan Hospital, Jinan, China; ^4^ Division of Clinical Pharmacology, Children’s National Hospital, Washington, DC, United States; ^5^ Departments of Pediatrics, Pharmacology & Physiology, Genomics & Precision Medicine, The George Washington University School of Medicine and Health Sciences, Washington, DC, United States; ^6^ Department of Paediatric Pharmacology and Pharmacometrics, University of Basel Children’s Hospital, Basel, Switzerland; ^7^ Department of Internal Medicine, Third Hospital of Hebei Medical University, Shijiazhuang, China

**Keywords:** meropenem, high dose regimen, carbapenem-resistant enterobacteriaceae infection, model-based therapeutic drug monitoring, preterm neonate

## Abstract

Carbapenem-resistant enterobacteriaceae (CRE) bloodstream infections have been rapidly spreading worldwide with a high mortality and pose a challenge to therapeutic decision-making, especially in premature neonates because insufficient empirical antimicrobial therapy is independently associated with high mortality. This case reported that a premature infant with CRE bloodstream infection was treated successfully with high-dose meropenem treatment with model-based therapeutic drug monitoring (TDM). In clinical settings, treatment target attainment of meropenem can be improved by increasing the frequency of administration, prolonging the infusion time, and using a high dose. This case report shows a successful regimen for CRE infection in a premature neonate and emphasizes the utility of model-based TDM of high-dose meropenem treatment. The adequate antimicrobial benefit provided by innovative techniques could ensure the efficacy and safety of high-dose meropenem therapy for CRE infection.

## Introduction

Carbapenem-resistant enterobacteriaceae (CRE) bloodstream infections have been rapidly spreading worldwide with a high mortality rate of about 30–70% (Tumbarello et al., 2012). One of the most frequent CRE pathogens is *K. pneumoniae*. Carbapenems have been used successfully for the treatment of severe infections with sensitive Gram-negative bacteria (Fritzenwanker et al., 2018). However, the prevalence of CRE poses a challenge to therapeutic decision-making, especially in premature neonates. The optimal antimicrobial therapeutic regimen for CRE bloodstream infections is still a matter of debate in clinical practice, but it is clear that insufficient empirical antimicrobial therapy is independently associated with high mortality (Tumbarello et al., 2012). Several retrospective studies have reported that in adult patients improved outcomes could be achieved with a combination therapy of carbapenems and other antibiotics (Tumbarello et al., 2012; Giannella et al., 2018) along with higher doses and/or prolonged infusion strategies (Giannella et al., 2018). However, there is still hardly any experience in neonatal clinical practice.

## Case Description

A neonate born prematurely at 27 weeks’ gestation with a birth weight of 970 g was admitted to the neonatal intensive care unit. Apgar scores were 5, 7, and 7 at 1, 5, and 10 min. On arrival, clinical examination did not show any abnormal findings, and the neonate had an unremarkable clinical course during the first 2 weeks of life with normal repeated clinical and laboratory evaluations.

On the 15^th^ day of life the neonate developed signs of neonatal sepsis based on clinical signs including oxygen fluctuations and abdominal distension and a clearly abnormal laboratory work-up that showed metabolic acidosis (blood pH 7.15) (normal range: 7.35–7.45) and several abnormal inflammatory indices such as elevated procalcitonin (PCT) concentration (12.6 ng/ml) (normal range:<0.5 ng/ml), C-reactive protein (CRP) (14.8 mg/l) (normal range: <8 mg/L), white blood cell count (WBC) (2.19 × 10^9^/L) (normal range: 5 × 10^9^/L–20 × 10^9^/L), and platelets (PLT) (51 × 10^9^/L) (normal range: 100 × 10^9^/L–300 × 10^9^/L). Antibacterial treatment was started immediately and consisted of meropenem (20 mg/kg, q12h) and vancomycin (15 mg/kg, single dose). On the same day neonatal sepsis with Gram-negative bacteria was confirmed by blood culture using BacT/Alert 3D Blood Culture Systems, and also a rapid increase in levels of PCT (116.1 ng/ml) and CRP (54 mg/l) was seen. Cerebrospinal fluid could not be obtained due to the neonate’s poor clinical condition and intolerance to the attempted lumbar puncture. Nevertheless, neonatal meningitis was suspected and the meropenem dose was increased from 20 to 40 mg/kg; meanwhile, vancomycin was discontinued. A high-dose meropenem regimen (40 mg/kg, q12h) was given off-label by intravenous infusion over 30 min for a week (days 15 to 21). In order to monitor the effectiveness of treatment and avoid occurrence of adverse reactions, model-based therapeutic drug monitoring (TDM) was performed. The covariate values of albumin and serum creatinine were 33.8 g/L and 80 μmol/L, respectively. The sample for TDM was obtained using an opportunistic sampling approach before and during the high-dose meropenem treatment. The concentration of meropenem was measured by high performance liquid chromatography (Sun et al., 2011). A previously reported population pharmacokinetic model was used to calculate the time of free drug concentration exceeding the minimal inhibitory concentration (fT > MIC) (Smith et al., 2011).

**Figure 1:**
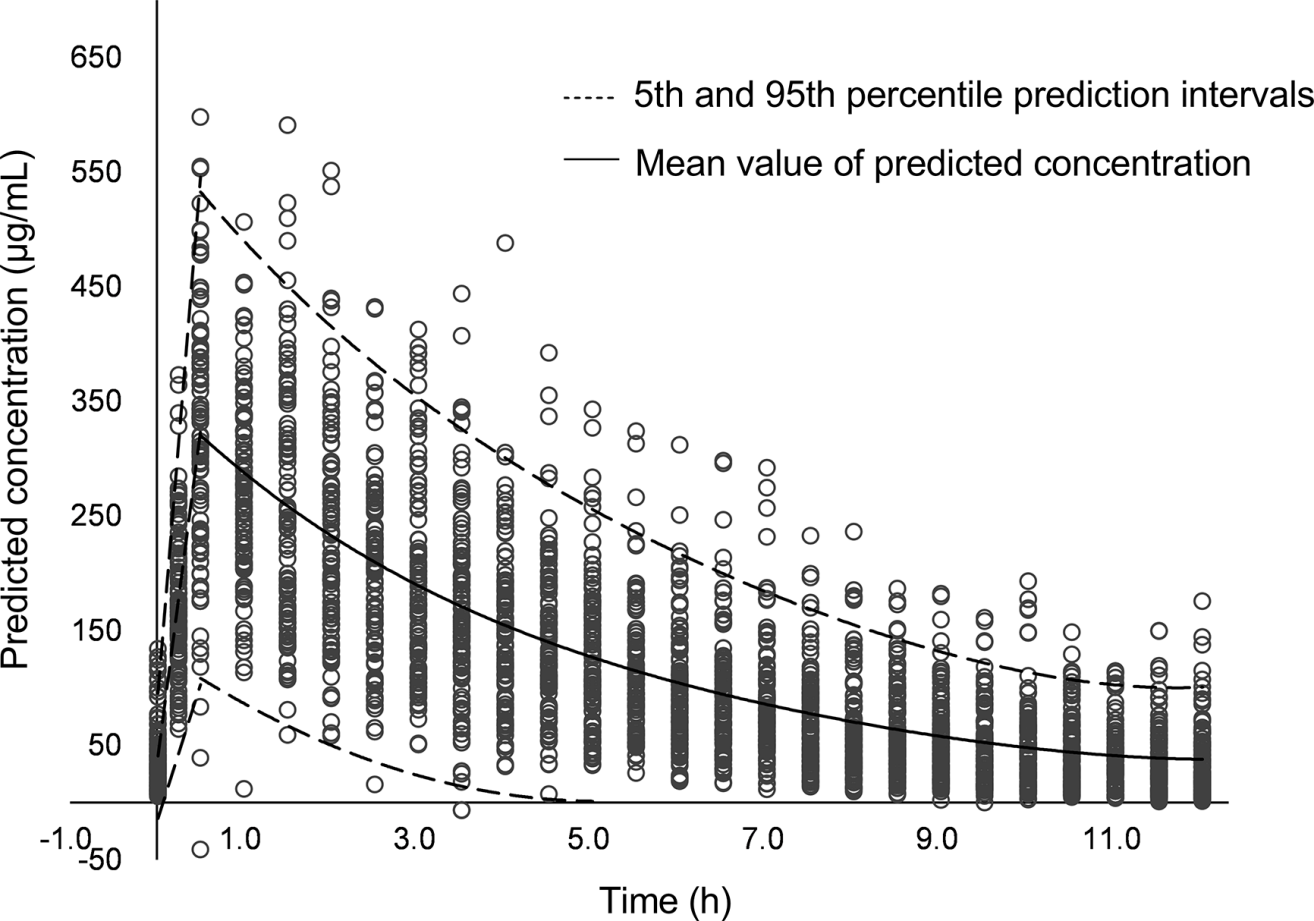
The simulated pharmacokinetics profile with 95% confidence intervals after giving the high-dose meropenem.

**Figure 2:**
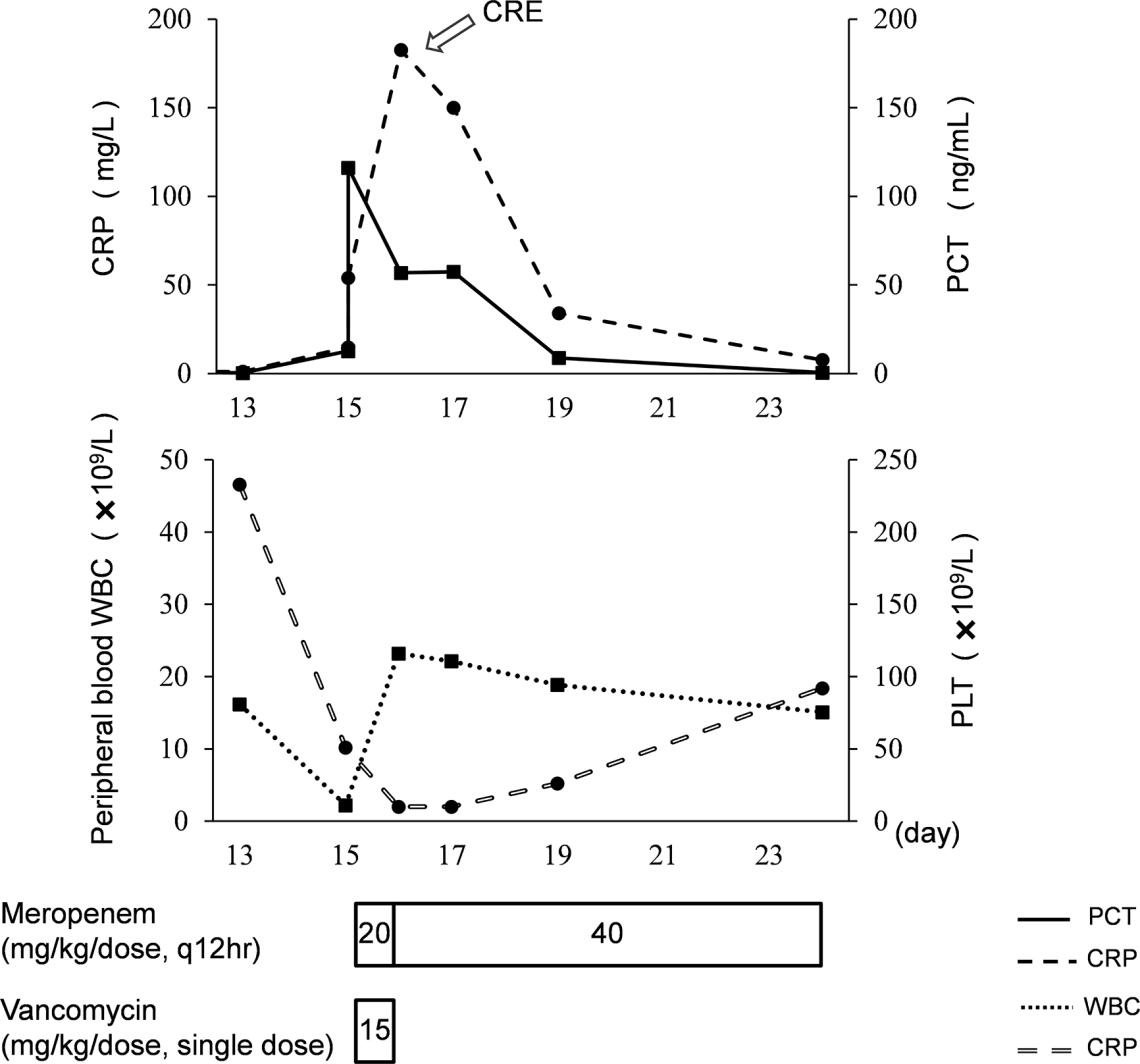
Clinical course of the premature neonate with CRE infection. PCT, procalcitonin; CRP, C-reactive protein; WBC, white blood cell count; PLT, platelet.

On day 18, an antimicrobial susceptibility test (AST) was conducted using VITEK^®^ 2 system after 3 days’ blood culture, and the results of AST showed *K. pneumoniae* resistant to carbapenems (MIC 8 mg/L), indicating CRE infection. The isolate was also resistant to other antibiotics, such as imipenem, piperacillin/tazobactam, cefoperazone/sulbactam, cefotaxime, ceftazidime, aztreonam, ertapenem, and cefuroxime. The isolate was sensitive to levofloxacin and amikacin, but the use of fluoroquinolones and aminoglycosides is not allowed for use in neonates in China. The meropenem concentrations of samples obtained 0.4 h (day 15) and 0.9 h (day 19) post dose were 37.9 and 26.6 ug/ml, respectively. The model-based pharmacokinetic–pharmacodynamic analysis by NONMEM software according to the population pharmacokinetic model of meropenem in premature and term infants reported by *P. Brian* et al. (Smith et al., 2011) showed that this patient with CRE infection had 53% fT > MIC when given 20 mg/kg meropenem on day 15 and 72% fT > MIC when given 40 mg/kg on day 19. Using 70% fT > MIC (the drug concentration was above the MIC during 70% of the dosage interval) as the pharmacokinetic–pharmacodynamic target, the probable target attainment was 99.2% by giving the high-dose regimen after 1,000 Monte Carlo simulations. The simulated pharmacokinetics profile with 95% confidence intervals after giving the high-dose regimen is presented in **Figure 1**. The high-dose regimen ensured acceptable pharmacokinetic–pharmacodynamic target for CRE infection. Subsequently, with the treatment of high-dose meropenem, the condition of the baby improved with normalization of PCT, CRP, WBC, and PLT levels (**Figure 2**) and negative blood culture. The cerebrospinal fluid was examined on day 20, and it showed no obvious abnormality [WBC: 10 × 10^6^/L (normal range: 0 × 10^6^/L–29 × 10^6^/L), protein: 1.56 g/L (normal range: 0.65–1.5 g/L), glucose: 2.35 mmol/L (normal range: 1.344–3.53 mmol/L)]. Adverse effects possibly due to meropenem were not observed during the treatment. Written informed consent was obtained from the minor’s legal guardian/next of kin for the publication of any potentially identifiable images or data included in this article.

## Discussion

This case reported that a premature infant with CRE bloodstream infection was treated successfully by high-dose meropenem treatment with model-based TDM. Insufficient empirical antimicrobial therapy is independently associated with high mortality (Tumbarello et al., 2012). In clinical settings, treatment target attainment of meropenem can be improved by increasing the frequency of administration, prolonging the infusion time, and using a high dose (van den Anker et al., 2009). This case report shows a successful regimen for CRE infection in a premature neonate and emphasizes the utility of model-based TDM of high-dose meropenem treatment. Premature neonates with underlying disease such as chronic lung disease, esophageal atresia, and congenital heart disease are extremely vulnerable who need more precise medication to avoid inadequate or excessive drug exposure. The approach of model-based TDM of drugs can directly reflect the drug exposure levels in patients by integrating drug concentrations, ontogenetic factors, and laboratory test results, which can extrapolate to premature infants mentioned above. The adequate antimicrobial benefit provided by innovative techniques could ensure the efficacy and safety of high-dose meropenem therapy for CRE infection.

## Data Availability Statement

The raw data supporting the conclusions of this article will be made available by the authors, without undue reservation, to any qualified researcher.

## Ethics Statement

The studies involving human participants were reviewed and approved by the Medical ethics committee of The First Affiliated Hospital of Shandong First Medical University. Written informed consent to participate in this study was provided by the participants' legal guardian/next of kin. Written informed consent was obtained from the minor's legal guardian/next of kin for the publication of any potentially identifiable images or data included in this article.

## Author Contributions

WZ and X-YC coordinated and supervised data collection and critically reviewed and revised the manuscript. Y-EW performed TDM and data analysis and drafted the initial manuscript. H-YX and H-YS managed the patient, collected the clinical data, and reviewed the manuscript. JA provided advice, critically reviewed and revised the manuscript. All authors contributed to the article and approved the submitted version.

## Funding

This work was supported by the Young Research Programme of the Health and Family Planning Commission of Hebei Province [grant number 20150275] and course development grants [grant numbers QLYXJY-201755, SDYAL17006, 2016A10, SDYY18008] from Shandong University and Shandong Province.

## Conflict of Interest

The authors declare that the research was conducted in the absence of any commercial or financial relationships that could be construed as a potential conflict of interest.
